# Group and Spend: An Evaluation of Perioperative Group and Save Practices in Traumatic Forearm, Wrist, Hand, Foot and Ankle Fracture Fixations and Their Associated Costs

**DOI:** 10.7759/cureus.74009

**Published:** 2024-11-19

**Authors:** Calvin R Finlayson, Steven W Hamilton

**Affiliations:** 1 Trauma and Orthopaedics, Aberdeen Royal Infirmary, Aberdeen, GBR

**Keywords:** direct cost, foot and ankle trauma, group and save, open reduction and internal fixation (orif), orthopaedics, pre-operative management, trauma, wrist fracture

## Abstract

Background

Fractures of the peripheral limbs make up a significant proportion of the caseload seen by an Orthopaedic Department. Some of these fractures will require surgical intervention and typically undergo open reduction and internal fixation (ORIF). Current guidance states that patients undergoing such procedures do not require group and save testing prior to theatre. Despite this, many patients still undergo these tests, which are seldom utilised to facilitate intraoperative or postoperative transfusion.

Aim

This article sets out to determine the incidence of group and save testing performed within a tertiary trauma service, as well as the rate of transfusions observed and any potential relationship with pre-operative haemoglobin as a predictor of transfusion. The financial cost of performing group and save tests, outwith current guidance, will also be determined to assess the financial impact on the trust.

Methodology

A three-month retrospective cohort analysis was conducted, utilising theatre planning records from June 1, 2024, to August 31, 2024, to identify patients undergoing single-procedure ORIFs of the forearm, wrist, hand, ankle or foot. Each patient’s electronic patient record was then examined to determine age, sex, pre-operative haemoglobin concentration, number and date of group and save tests, and whether they were issued or received blood products. The individual cost of one group and save test was found through enquiry with the local blood transfusion laboratory as £10.77.

Results

There were 117 patients who underwent 118 distal limb ORIFs and a total of 105 group and save samples sent. The mean pre-operative haemoglobin was 132.5 g/L, with a minimum observed haemoglobin of 94 g/L. No patient received blood products intraoperatively or postoperatively. The total cost of group and save testing in the period observed was found to be £1,130.85. The projected annual cost of the group and save testing for this cohort was £4,523.40.

Conclusion

This study finds that, despite local and national guidance, a significant number of group and save tests are being conducted unnecessarily, at significant cost to the trust. There is no relationship observed between pre-operative haemoglobin and transfusion requirement, which confirms that current guidance is appropriate. Increasing adherence to current policy is projected to save the trust up to £4,523.40 annually. It is recommended that other centres audit their use of pre-operative testing for appropriateness, utility and cost.

## Introduction

In the United Kingdom, fractures of the forearm, wrist, hand, ankle and foot account for a significant proportion of trauma cases - up to as much as 69.7% of a trauma unit’s caseload [1] - and have a reported incidence of approximately 51.8 per 10,000 population [2]. Where these cases require surgical management, it is important to ensure a thorough pre-operative assessment takes place, in which considering the potential for blood loss and transfusion plays a role.

Performing a group and save blood test is a requirement in order to facilitate transfusion. Current National Institute for Health and Care Excellence (NICE) guidance on complex and non-complex fractures and routine elective pre-operative investigations make no recommendation on performing such tests [3-5]; however, local policies may vary. By performing these tests, a patient’s ABO group and Rhesus status are determined to facilitate the transfusion of appropriately matched blood products when required, such as for intraoperative blood loss or supportive transfusions in the pre- or post-operative period. This matching process reduces the risk of ABO incompatibility, which can have severe consequences, such as intravascular haemolysis [6]. In the event of an emergency transfusion being required, most centres can make use of either a major haemorrhage protocol or emergency type O negative blood stocks kept in close proximity to the surgical theatre as part of the management of severe acute haemorrhage. While this can be accessed in an emergency, the supply of blood can fluctuate nationally [7]. While type O negative is preferentially used for emergencies due to lacking immunogenic surface proteins, other ABO types are available, which are typically mobilised after the patient’s blood type is known. This process typically requires two blood samples from the patient, with an identical ABO typing result in each, to prevent the issue of incompatible products.

In the conduct of surgery, a degree of haemorrhage is always expected, and having the patient’s ABO status known and ready for the mobilisation of blood products could reduce the time required to initiate a transfusion in the peri-operative setting. Common occasions when patients might be transfused include treating traumatic anaemia pre-operatively or in the post-operative setting to counter blood loss.

While pre-emptively performing a group and save is current local practice in NHS Grampian for trauma procedures such as hip arthroplasty, a number of papers have examined whether these tests are still required in other surgical procedures, such as laparoscopic appendicectomy and mastectomies [8-12], and found these to be unnecessary for some procedures. By comparison, there is little literature for trauma and orthopaedic services with regard to whether such testing is required in acute trauma for those with peripheral extremity fractures undergoing fixation. Typically, procedures such as open reduction and internal fixation (ORIF) of the peripheries make use of a surgical tourniquet as a means to achieve haemostasis and minimise intraoperative bleeding [13]. This is of dual benefit: both to the patient, by way of reducing the need for post-operative transfusion of blood or crystalloids for blood pressure support, and to the surgeon, by minimising bleeding into and hence obscuring the surgical field. However, there remains an intraoperative risk of injury to significant vascular structures, such as the radial and ulnar arteries of the hand, and the anterior and posterior tibial arteries of the foot. As these procedures are often minimally haemorrhagic, this study sets out to evaluate whether this necessitates the use of group and save testing and, if this can be avoided, what financial impact this may have on healthcare organisations. In doing so, the relationship between pre-operative haemoglobin concentration will be examined to determine if this is a predictor of transfusion requirement, and the number of group and save samples processed per patient will be examined to determine the financial burden of this.

## Materials and methods

A three-month retrospective cohort study was performed by analysing theatre records from Bluespier Patient Manager software between June 1, 2024, and August 31, 2024, containing all trauma procedures conducted in Aberdeen Royal Infirmary or those transferred onward to Woodend General Hospital to enhance surgical capacity. Of these, 578 procedures were identified prior to screening with pre-determined inclusion and exclusion criteria. This study included those undergoing single-procedure ORIFs involving the radius, ulna, any bone of the hand, distal tibia, distal fibula or any bone of the foot in patients aged 16 or over. Any procedure carried out on, or more proximally to, the distal humerus or tibial shaft was excluded. Patients who required activation of the major haemorrhage protocol on admission, underwent multiple procedures in one theatre session or underwent any procedure not recorded via Bluespier were also excluded.

Of the subsequently screened 117 patients, their electronic patient records (EPRs) were examined to determine the incidence of pre-operative group and save sampling and blood product issuance, in addition to their latest pre-operative haemoglobin, within one month of the procedure, where recorded. Recorded group and save samples included those taken during any prior clerking admission, within one month of the procedure and those taken during the operative admission. To determine the incidence of expired samples, any sample taken over 72 hours prior to surgery was recorded as expired to reflect the local Blood Transfusion Service (BTS) laboratory policy.

This data was collated in Microsoft Excel (Microsoft® Corp., Redmond, WA, USA) before analysis was conducted, including one-way analysis of variance (ANOVA). A threshold of less than 130 g/L haemoglobin concentration was established to define pre-operative anaemia, in line with the current guidance from the British Society for Haematology [14]. The cost of performing one group and save test was determined as £10.77 through enquiry with the local BTS laboratory.

## Results

In total, 117 patients underwent a total of 118 ORIFs of the forearm, wrist, hand or foot across two sites. The demographics of this population are demonstrated in Table 1. The mean monthly procedure rate was 39 procedures per month, with an incidence between 20.3% and 21.2% of the total trauma cases seen each month.

**Table 1:** Demographics and procedure counts ORIF, Open reduction and internal fixation

Total	Count
Procedures	578
Suitable ORIFs	118
Total patients	117
No. of males	42
No. of females	75
Median age	60

The incidence of group and save testing, pre-operative haemoglobin analysis and transfusion practices can be seen in Table 2. Across all included procedures, group and save testing was performed on 63 patients a total of 105 times, with a mean incidence of 35 tests per month. Of the 105 tests, 36 were conducted over 72 hours pre-procedure and were expired before surgery.

**Table 2:** Testing incidence, haemoglobin analysis and transfusion practices G&S, Group and save; RBC, Red blood cell

Analysis	Month			Total	Monthly mean
	June	July	August		
Patients tested	22	19	22	63	21
Percentage of patients tested (%)	57.9	48.7	53.7	53.8	---
Total G&S samples	42	29	34	105	35
Total G&S >72 hours pre-operative	15	2	19	36	12
Pre-operative haemoglobins performed	33	37	35	105	35
Pre-operative haemoglobins unavailable	5	2	6	13	4
Mean pre-operative haemoglobin	129.7	136.8	131.1	132.5	---
Median pre-operative haemoglobin	128	138	134	134	---
No. of pre-operative haemoglobin <130	18	11	16	45	15
Minimum haemoglobin per month	94	101	97	---	97.3
Total units RBC issued	3	2	1	6	2
Total units RBC transfused	0	0	0	0	0

A total of 105 pre-operative haemoglobins were checked, while 13 procedures did not have a pre-procedure haemoglobin. Of the 105 checked haemoglobins, the mean haemoglobin was found to be 132.5 g/L, with 45 (42.8%) of these under 130 g/L, the threshold for detecting pre-operative anaemia. The minimum haemoglobin concentration recorded was 94 g/L. In examining the subsequent transfusion practices, five patients were issued a total of six units of packed red blood cells from the local blood bank. Of these issued blood products, none were given to any patient, as recorded on their EPR.

The unit price of performing one group and save test within the local blood transfusion laboratory was £10.77. Over the three-month period examined, the total cost of testing was determined to be £1,130.85, with an average monthly cost of £376.95. The estimated annual cost of group and save testing in this unit is, therefore, £4,523.40. Of the total 36 expired samples taken, the cost was £387.72 over the duration of the review period, representing 34.3% of the total cost observed. These costs are detailed in Table 3.

**Table 3:** Costs of group and save test

Time period		Cost
June	Valid	290.79
	Expired	161.55
July	Valid	290.79
	Expired	21.54
August	Valid	161.55
	Expired	204.63
June-August 2024		1130.85
Estimated annual cost		4523.40

A one-way ANOVA test determined that there was no significant difference in the number of group and save samples between all months (p = 0.23). There was no correlation between pre-operative haemoglobin and red blood cell transfusions delivered.

## Discussion

This research affords the opportunity to evaluate the necessity and value of performing pre-operative group and saves for both the patient and surgeon. While this has been explored in some regards by general surgery and plastic surgery, there is relatively little in the literature from a trauma and orthopaedics standpoint regarding ORIF procedures. The current orthopaedic literature has investigated shoulder, hip and knee arthroplasty, finding similar results, with low numbers of patients requiring post-operative transfusion. Hainsworth et al. [12] determined that arthroplasty transfusion rates can vary from 9.9% for hip arthroplasties to 3.8% for knee arthroplasties. By comparison, this study has seen a transfusion rate of 0%.

There are a variety of tools available to surgeons to secure haemostasis and reduce blood loss, such as tourniquets, diathermy, antifibrinolytics and cell salvage, but the use of these techniques was not analysed. These practices may vary according to procedure and surgeon, and exploring their employment and potential relationship to observed blood loss may be a further research avenue. This study may also be impacted by confounders, such as selection bias, where patients with poor physiological reserve are more likely to be managed conservatively, skewing the population analysed towards those more favourable for surgery. Another potential confounder is instances where patients are admitted with polytrauma, where other injuries are managed conservatively, and peripheral fractures undergo fixation as described previously. In such cases, these patients are rightly group and saved, but they were not identified or excluded by the methodology. As mentioned, these patients typically require at least two identical results on group and save tests before blood can be issued from the BTS laboratory. In this study, it is assumed that all patients have met this requirement preoperatively, as such information can only be referenced using the national BTS database, which was unavailable to the author at the time of analysis. Therefore, it is possible that some patients would require further samples to meet the two-sample policy, thus increasing the overall cost, as well as the percentage of "wasted" tests, as these single samples are not valid for requesting a transfusion. Additionally, the number of refused tests has not been factored into the total cost of group and save testing, as this is not recorded in the patient's EPR. These would also factor into the number of waste tests, but would not increase the processing cost, as the samples would not be tested. There are other costs, such as heating, lighting, equipping and staffing the laboratory, as well as the costs of performing venepuncture, which are not included in these totals.

While this study makes use of a relatively small time period, it has produced a higher number of monthly cases than other papers, such as Magowan et al. [9]. Expanding the retrospective analysis to 12 months would increase the available data and allow a comparison of the expected annual cost to the determined annual cost. The local department has implemented a new tool to aid clinicians in deciding which procedures are appropriate for performing group and save tests, which may influence future numbers of group and save tests performed.

## Conclusions

This study concludes that current guidance remains accurate and that there is no indication to perform group and save tests on trauma patients undergoing surgical fixation of any fracture of the forearm, wrist, hand, ankle or foot. There was no relationship observed between pre-operative haemoglobin concentrations and transfusion requirements. Had local guidance been followed, the local trust could have saved £1,130.85 during the three-month review period. It is projected that the trust could save up to £4,523.40 annually if such tests were not performed and current guidance is followed. It is recommended that other units also audit their own peri-operative testing to ensure that the tests being conducted are relevant and beneficial to both the patient and their own hospital trust.
